# Influences of Medical Crowdfunding Website Design Features on Trust and Intention to Donate: Controlled Laboratory Experiment

**DOI:** 10.2196/25554

**Published:** 2021-05-04

**Authors:** Xing Zhang, Wenli Hu, Quan Xiao

**Affiliations:** 1 School of Management Wuhan Textile University Wuhan China; 2 School of Information Management Jiangxi University of Finance and Economics Nanchang China

**Keywords:** medical crowdfunding, website design, cognition-based trust, affect-based trust, intention to donate

## Abstract

**Background:**

As a type of donation-based crowdfunding, medical crowdfunding has gradually become an important way for patients who have difficulty paying medical bills to seek help from the public. However, many people still have limited confidence in donating money to medical crowdfunding projects.

**Objective:**

Given that the features of a medical crowdfunding website may be important to gain users’ trust, this study draws upon two-factor and trust theories to explore how different design features of medical crowdfunding websites affect potential donors’ cognition-based trust and affect-based trust, and how these types of trust affect the intention to donate.

**Methods:**

A 2 (informativeness: high vs low) × 2 (visual cues: cool color vs warm color) × 2 (social cues: with vs without) between-subject laboratory experiment was performed to validate our research model. A total of 320 undergraduate students recruited from a university in China participated in the controlled laboratory experiment.

**Results:**

Cognition-based trust (β=.528, *P*<.001) and affect-based trust (β=.344, *P*<.001) exerted significant effects on the intention to donate of potential donors of medical crowdfunding. Informativeness as a hygiene factor positively influenced potential donors’ cognition-based trust (*F*_1,311_=49.764, *P*<.001) and affect-based trust (*F*_1,311_=16.093, *P*<.001), whereas social cues as a motivating factor significantly influenced potential donors’ cognition-based trust (*F*_1,311_=38.160, *P*<.001) and affect-based trust (*F*_1,311_=23.265, *P*<.001). However, the color of the webpages affected the two dimensions of trust differently. Specifically, medical crowdfunding webpages with warm colors were more likely to induce affect-based trust than those with cool colors (*F*_1,311_=17.120, *P*<.001), whereas no significant difference was found between the effects of cool and warm colors on cognition-based trust (*F*_1,311_=1.707, *P*=.19).

**Conclusions:**

This study deepens our understanding of the relationships among the design features of medical crowdfunding websites, trust, and intention to donate, and provides guidelines for managers of medical crowdfunding platforms to enhance potential donors’ trust-building by improving the website design features.

## Introduction

### Background

Medical expenses are forcing a staggering number of people into poverty worldwide [1]. According to data from the US Census Bureau, 11.2 million Americans are living below the poverty line because of health care costs [2]. Although public health insurance provides coverage for almost all of China’s 1.4 billion people, many patients and their families still have difficulty paying medical bills that outpace government insurance provision [3]. Some of them have had to take out loans to pay for their medical expenses, adding to their financial woes and imposing a growing burden of consumer debt on China [4]. As a result, medical crowdfunding, a convenient and effective way to raise funds to alleviate the burden of medical expenses, has skyrocketed in the last few years [5]. For example, GoFundMe, a world-leading crowdfunding platform, reports that more than one-third of the fundraising campaigns it hosts are for health care expenses and that these campaigns raise more than US $650 million annually [6].

In the context of charitable giving, the information asymmetry that exists between charitable project promoters and potential donors is a barrier to successfully soliciting individual donations. Potential donors are often deprived of complete (and updated) information about how their donations will be used [7]. They need charitable project promoters to disclose information about their past performance and governance, as well as information about beneficiaries [8]. To adequately address the information asymmetry problem, and thus increase donations, promoters need to share customized information with targeted donors through appropriate information channels. Medical crowdfunding platforms help patients to make personalized charitable appeals, and to show their illnesses and financial difficulties on their own webpages, which can partially reduce the level of information asymmetry between the crowdfunders and the donors. As such, medical crowdfunding is becoming increasingly popular as it harnesses the power of the crowd to raise funds and reduce the risk of “medical bankruptcy” [5].

Despite the popularity of medical crowdfunding, the failure rate of crowdfunded projects is reported to be high. Many promising projects have failed to meet their fundraising goals for different reasons. The lack of experience in the use of information technology (eg, computers and the internet), insufficient health care literacy, and poor writing skills can all contribute to the failure of a medical crowdfunding project [9]. Fraudulent campaigns, loss of privacy, and small personal social networks may be additional barriers to receive donations from others [9,10]. Hence, many studies have focused on identifying the factors that influence the success of charitable crowdfunding, such as donors’ intrinsic and extrinsic motivation [11,12], project content [13], project goal [14], fundraisers’ characteristics [13-15], and social influence [16].

Recently, several studies have recognized trust as a critical determinant of funding intention in different crowdfunding contexts [17-20]. In the context of medical crowdfunding, many people have limited confidence in contributing to crowdfunding projects because they are afraid of fraud or do not know whose needs are more urgent. These trust issues result in many medical crowdfunding projects being not fully funded. Therefore, focusing on how to increase people’s trust in medical crowdfunding platforms and projects is important. Medical crowdfunding is similar to crowdfunding based on rewards or equity; however, it also has unique characteristics. Medical crowdfunding can essentially be considered as an act of philanthropy, and its success requires not only rationally convincing potential donors but also emotionally motivating them. However, the existing literature on other types of crowdfunding have primarily focused on the effect of a single rational dimension of trust, cognition-based trust [20,21], while largely neglecting the effect of the emotional dimension of trust, affect-based trust. Previous research has noted that emotional appeals are strongly associated with prosocial behavior [22]. Therefore, the first aim of this study was to investigate the role of affect-based trust in a medical crowdfunding context and to examine how such trust affects the intention to donate simultaneously with cognition-based trust.

Despite the increasing number of studies on the relationship between trust and crowdfunding investments, less attention has been paid to the factors that build trust in the context of medical crowdfunding. Therefore, the second focus of this study was on the antecedents of cognition-based and affect-based trust in medical crowdfunding, which can help us to better understand the development of trust. Among the limited research on the antecedents of trust in the field of crowdfunding and charitable giving, most studies have focused on the project type, project content, and fundraisers’ expertise and reputation [18-20], whereas few studies have considered how website design features affect potential donors’ trust in crowdfunding platforms and projects. Medical crowdfunding websites play an especially important role in publicizing projects and persuading people to donate. On medical crowdfunding websites, fundraisers can write their own charitable appeals to describe their personal experiences of illness and financial difficulties to attract potential donors. As such, medical crowdfunding platforms need to devote considerable efforts to their website design to increase trust and facilitate online donations. Thus, research on design features of medical crowdfunding websites is needed to understand the role of the medical crowdfunding website in influencing people’s trust and intention to donate.

To bridge the aforementioned research gaps, in this study, we adopted two-factor and trust theories to identify hygiene and motivation factors in the website design of a medical crowdfunding website, and examined how they affect people’s cognition-based trust and affect-based trust, and their ultimate intention to donate.

### Trust

#### Trust in Medical Crowdfunding

The rapid growth of crowdfunding platforms has attracted the attention of scholars, and numerous studies have been performed on the factors that influence crowdfunding success. In these studies, trust has emerged as a key determinant of funding intention in different crowdfunding contexts [17-20]. Trust can be defined as a willingness to accept vulnerability based on beliefs about the trustee’s ability and character, and the emotional bond between the trustor and the trustee [23]. This definition indicates that trust includes not only the intellectual/cognitive dimension (ie, cognition-based trust) but also the emotional/affective dimension (ie, affect-based trust [24]). Table 1 presents a sampling of quantitative research on the role of trust in crowdfunding, revealing the relatively minimal research that has focused on the context of donation-based crowdfunding. Moreover, these studies have provided only partial insight into the two dimensions of trust in crowdfunding. Most of them have conceptualized trust as a one-dimensional construct and revealed that trust in the crowdfunding platform or project creators has a positive effect on individuals’ investment decisions [25]. Some other scholars have primarily addressed donors’ cognition-based trust, arguing that the credibility and the trustworthiness of the fundraiser and platform are vital factors that influence investors to fund the project [26]. However, little attention has been devoted to the cognitive and affective dimensions of trust simultaneously in the context of crowdfunding.

Similar to other types of crowdfunding, potential donors of medical crowdfunding need to scrutinize the quality of the projects and judge their credibility before deciding to donate. In addition, medical crowdfunding can be regarded as an act of philanthropy where potential donors are incentivized to donate out of sympathy for specific patients seeking help. Therefore, the emotional dimension of trust is also noteworthy because it can evoke an emotional response and inspire people to donate. Hence, this study attempts to contribute to trust theory and the crowdfunding literature by examining whether cognition-based trust and affect-based trust exert positive effects on intention to donate in the context of medical crowdfunding.

**Table 1:** Representative previous research on the role of trust in crowdfunding.

Reference	Context	Dependent variable	Trust-related variable
Zhang et al [27]	Donation-based crowdfunding	Actual donation	Platform trust
Behl et al [28]	Donation-based crowdfunding	Operational performance	Cognitive trust, swift trust
Kim et al [29]	Incentive crowdfunding	Willingness to crowdfund	Trust in platform, trust in fundraiser
Kim et al [12]	Incentive crowdfunding	Crowdfunding participation	Trust in platform, trust in fundraiser
Chen et al [25]	Donation-based crowdfunding	Intention to donate	Trust
Wehnert et al [30]	Crowdfunding	Trust	Trust
Strohmaier et al [21]	Reward-based crowdfunding	Pledging intention	Trust in creators, trust in the platform
Liang et al [19]	Reward-based crowdfunding	Investment intention	Funder’s trust
Cascino et al [26]	Reward-based crowdfunding	Consumer protection regulation, project funding	Perceived credibility of disclosure
Rodriguez-Ricardo et al [31]	Crowdfunding	Intention to participate in crowdfunding	Trust in crowdfunding
Yang et al [32]	Crowdfunding	Investment intention	Trust
Jones and Moncur [33]	Reward-based crowdfunding	Likelihood to invest	Funder’s trust
Xiao [34]	Equity-based crowdfunding	Investment intention	Competence trust, relational trust
Klement and Teubner [35]	Equity-based crowdfunding	Number of investments	Trustworthiness

#### Cognition-Based Trust, Affect-Based Trust, and Intention to Donate

Generally, cognition-based trust is grounded in careful and rational reasoning [36]; it focuses on the “good reasons” why individuals are trustworthy and calls for rational choices based on credible information about the intentions or capabilities of others [37]. Previous research has found that potential sponsors often depend on their evaluation of a crowdfunding project’s credibility when they decide whether or not to donate their money to the cause [15,38]. In the field of medical crowdfunding, if potential donors believe that the crowdfunding platform is reliable and the charitable appeal of the fundraiser is logical and reasonable, then their cognition-based trust will be promoted, thereby increasing their intention to donate. Thus, we propose the following hypothesis:

H1: Potential donors’ cognition-based trust is positively correlated with intention to donate.

By contrast, affect-based trust focuses on the “emotional bond” between parties. This type of trust is derived not from one’s understanding and reasoning but rather from their instincts and feelings [23,37]. Kang et al [18] provided evidence that investors’ trust related to emotions has a positive effect on their willingness to invest in crowdfunding platforms. Liu et al [15] also revealed that individuals will increase their willingness to donate because of their affective state derived from the concerns of the charitable crowdfunding fundraiser’s situation. Patients can use crowdfunding platforms to connect emotionally with potential donors to raise funds for health care [39]. Thus, we propose the following hypothesis:

H2: Potential donors’ affect-based trust is positively correlated with intention to donate.

Previous studies have argued that individuals will invest further in a relationship only when their basic expectations about the reliability of the relationship are met [24]. Cognition-based trust influences affect-based trust, particularly in the beginning of relationship formation [23]. In many online contexts, cognition-based trust has been empirically demonstrated to be an antecedent of affect-based trust [23,40]. Kang et al [18] investigated the crowdfunding field and found that a rational assessment of the crowdfunding project allows the investor to decide whether to trust the fundraiser, which can enhance their communication and help establish a strong emotional connection. Xu and Wang [41] also argued that logically coherent and complete stories are a prerequisite for eliciting potential donors’ empathy and to inspire their charitable behavior. In the context of medical crowdfunding, the cognition-based trust that potential donors develop from carefully reviewing the information provided by the patients can reduce their feelings of insecurity, which in turn arouse emotional responses and facilitate a higher level of affect-based trust. Hence, we propose the following hypothesis:

H3: Potential donors’ cognition-based trust is positively correlated with their affect-based trust.

### Website Design Features and Trust

#### Antecedents of Trust in Crowdfunding

Considering that trust can be easily broken in virtual environments, some studies have been performed on how to establish and maintain trust in various contexts [42]. The building of trust in online communities has been wildly studied. For example, Mpinganjira [43] revealed that information usefulness, community responsiveness, and shared vision have a significant influence on consumers’ overall trust in health-related virtual communities. Nadeem et al [44] found that social presence of online brand communities, social presence of others, and social presence of interaction have positive effects on social commerce trust. Given that online communities are groups of people with common interests and shared goals, community members gradually develop trust in the community through long-term social interaction. Unlike online communities, people do not need long-term interactions to build trust in a crowdfunding context, but rather rely on a direct and rapid appraisal of the crowdfunding website and the project. Thus, understanding the antecedents of trust in the context of crowdfunding is critical in improving the success rate of crowdfunding projects [45].

Existing research in the field of crowdfunding has mainly examined three categories of antecedent variables of trust: project-, fundraiser-, and platform-related characteristics [15,18,20]. Table 2 provides examples of representative studies focusing on the antecedents of trust. Only a few studies investigated the platform-related characteristics that affect trust. Among these limited investigations, attention was mainly focused on the overall quality or functional features of the platform. For example, Liu et al [15] revealed that website quality and transaction convenience have positive effects on the perceived credibility of a charitable crowdfunding project. Kang et al [18] found that perceived accreditation, structural assurance, and a third-party seal of a crowdfunding platform exert different effects on investors’ trust beliefs. However, little attention has been paid to the question of how website design features affect individuals’ trust and intention to donate. Website design factors have been demonstrated to be critical in affecting user response and experience [46]. As people learn the details of funding projects by viewing medical crowdfunding websites, exploring the design features of medical crowdfunding websites may contribute to an improved appreciation of trust and intention to donate.

**Table 2:** Representative previous research on antecedents of trust in crowdfunding.

Reference	Context	Project-related characteristics	Fundraiser-related characteristics	Platform-related characteristics
Kim et al [29]	Incentive crowdfunding	Network externality, perceived informativeness	Value congruence, social interaction ties	Perceived accreditation, structural assurance, third-party seal
Strohmaier et al [21]	Reward-based crowdfunding	N/A^a^	Perceived monitoring	Perceived platform rules, perceived monitoring, perceived pledging security
Liang et al [19]	Reward-based crowdfunding	Project information quality	Fundraiser’s ability/expertise, fundraiser’s reputation	N/A
Rodriguez-Ricardo et al [31]	Crowdfunding	N/A	Altruism, internal locus of control	N/A
Yang et al [32]	Crowdfunding	Perceived benefits, perceived risk	Communication, shared value	N/A
Kang et al [18]	Reward-based crowdfunding	Network externality, perceived informativeness	Value congruence, social interaction ties	Perceived accreditation, structural assurance
Zheng et al [17]	Reward-based crowdfunding	N/A	Crowdfunding success experience, investment in others	N/A
Liu et al [15]	Donation-based crowdfunding	Project popularity, project content quality	Initiator reputation	Website quality (navigability, security, visual appeal), transaction convenience

^a^N/A: not applicable.

#### Website Design Features and Two-Factor Theory

A substantial body of literature in the field of information systems has investigated the relationship between website design features and trust. Several website design features have been identified as antecedent variables of trust in various contexts such as navigation design [47,48], privacy and security [47,49], website quality [50], and ease of use [46,48]. However, these antecedents have been examined in a disjointed manner and lack a holistic view of how website design features influence trust. Moreover, existing research has mainly identified the usability and functional features of websites from a cognitive perspective, with less consideration given to the emotional aspects of website design features [46]. Some researchers have demonstrated that a website that meets most usability guidelines does not necessarily lead to higher user favorability [51]. Other scholars have also argued that users’ evaluations of a website are often determined by their overall impression of the website, thereby requiring an examination of the holistic aspects of website design features [46,52]. Medical crowdfunding platforms not only need to cognitively enhance the design of their websites to persuade potential donors but should also harness the affective features of their website to evoke potential donors’ emotional response and facilitate their willingness to donate. Therefore, in this study, we adopted the Herzberg two-factor theory as a theoretical framework for integrating the cognitive and affective aspects of website design features that influence users’ trust in the context of medical crowdfunding.

The two-factor theory was proposed by Herzberg in 1959 [53], which is also known as the hygiene motivation theory. This theory was initially used to explain factors that lead to satisfaction or dissatisfaction in employees’ work environment and has gradually been used in a wide range of different domains [54]. Recently, an increasing number of studies have been performed using two-factor theory to examine website design features, and their effects on user attitudes and behaviors in different types of websites [55-57]. In these studies, website design features are systematically distinguished into two categories: hygiene and motivation factors.

#### Informativeness

Hygiene factors are a collection of attributes closely related to the basic functionality of a website [58]. As the primary purpose of a medical crowdfunding website is to provide the visitors with information about the crowdfunding project, informativeness was selected as the hygiene factor of a medical crowdfunding website in this study. Informativeness can be defined as the extent to which a website offers rich and useful information to users [59,60]; thus, it reflects a website’s ability to provide information to its visitors [61]. Previous research has demonstrated that website informativeness is an important prerequisite for users’ attitudes toward the website [62], and that it also plays a crucial role in reducing risk and building trust [63]. In the field of crowdfunding, few studies have explored the relationship between informativeness and trust. For example, Kang et al [18] found that funders’ cognition-based trust in crowdfunding projects is enhanced if the crowdfunding website provides funders with complete and timely information about the project through a bulletin board. In medical crowdfunding websites, greater informativeness (eg, charitable appeals with several texts and images) helps donors make rational judgments, which in turn makes it easier for them to develop cognition-based trust in the project and the platform. Therefore, we propose the following hypothesis:

H4a: Informativeness is positively correlated with potential donors’ cognition-based trust.

Previous research on shopping websites has revealed that the informativeness of a website positively influences consumers’ emotional responses [64]. Majumdar and Bose [13] also demonstrated that charitable crowdfunding fundraisers use detailed information to emphasize their needs and suffering to attract others’ attention. On the basis of emotional contagion theory, fundraisers’ suffering and sadness presented in their stories may increase potential donors’ affective reactions. Similarly, detailed information about a medical crowdfunding project not only gives the potential donor insight into the fundraiser’s situation but also serves as a means for the fundraiser to get others to listen and alleviate their own suffering. Adding words and images that describe the patient’s suffering can result in easily evoking potential donors’ affect response and enhancing their emotional bonding with the fundraiser. At the same time, rich project information allows potential donors to know more about the fundraiser and shorten the perceived psychological distance between them, thereby inspiring a higher level of affect-based trust. Hence, we propose the following hypothesis:

H4b: Informativeness is positively correlated with potential donors’ affect-based trust.

#### Visual Cues

Motivation factors are a collection of attributes that are closely related to the additional value-added services offered by a website [58]. Previous studies have found that in contrast to hygiene factors, motivation factors are crucial for website users’ satisfaction [55,57]. In the medical crowdfunding context, motivation factors are the website design features of a medical crowdfunding website that stimulate potential donors to make donation decisions beyond meeting their basic information needs. In this study, the website’s visual cues (ie, warm or cool colors) and social cues were selected as motivation factors.

The visual design of a website has proven to be an impactful determinant of trust [48]. Among the many visual design elements, color serves as a specific visual stimulus that shapes a visitor’s perception of the ambient temperature [65]. Such perceived cool or warmth on temperature elicits varying degrees of social proximity, language concreteness, and relational focus, and exerts an effect on individual behavior through the mechanism of insula [66]. Williams and Bargh [67] showed that the warmth sensed by the body can influence interpersonal judgments and prosocial behavior. Literature from several fields has noted that red and orange tend to evoke warmth, whereas blue and green are reminiscent of cold [68]; this comparison seems to convey a more positive role of warm colors. However, an ever-increasing number of studies in recent years have challenged this view by emphasizing the effects of cool colors. Rizomyliotis et al [69] found that cool backgrounds are more likely to inspire stronger positive attitudes and behavioral intentions than warm colors, and cool colors usually create a sense of relaxation and calm [70]. On the one hand, cool colors are often linked to competence, trust, safety, and sincerity, such that many companies incorporate blue into their brand logos to create a professional and trustworthy image, and consumers regard blue logos to be more reliable than other colors [71]. On the other hand, in terms of the information processing of individuals, Ettis [72] suggested that cool colors (eg, blue) facilitate individuals’ information seeking, processing, and consideration. Therefore, medical crowdfunding sites with more cool-colored elements may be more conducive to donor attention, and could promote rational and logical reasoning about the help-seeking information on the platform, which in turn will enhance the donors’ cognition-based trust in the platform and the project. Thus, we propose the following hypothesis:

H5a: Potential donors’ cognition-based trust in a medical crowdfunding website with a cool-toned (blue) interface is higher than that in a warm-toned (orange) interface.

Previous studies have documented that perceived warmth is associated with interpersonal intimacy; that is, perceived warmth creates feelings of interpersonal closeness [73], and promotes interpersonal trust, cooperation, and friendship [74]. Specifically, warm colors are more evocative of warmth than cool colors [66] and provide consumers with a sense that their surroundings are more socially dense [75]. In this manner, a warm-toned interface of a medical crowdfunding website will bring warm psychological perceptions and positive attitudes to donors, making them feel less distant from the medical crowdfunding platform and other help-seekers, and thus more likely to evoke their emotional trust toward the platform and project. Hence, we posit the following hypothesis:

H5b: Potential donors’ affect-based trust in a medical crowdfunding website with a warm-toned (orange) interface is higher than that in a cool-toned (blue) interface.

#### Social Cues

Social cues in web design refer to social presence and social interaction embedded in the web interface through various communication tools [76]. These cues have been shown to have important influences on individuals’ cognition-based trust in various contexts such as online shopping and e-commerce. For example, early consumer endorsement in the form of online reviews in e-commerce can help other consumers determine the usefulness of a product, build cognition-based trust, and thus facilitate rational shopping decisions [77]. Social cues can serve as a collective endorsement, allowing the crowdfunding fundraisers to signal credibility [78]. In medical crowdfunding, fundraisers need to reach out to others through social networks to attract more donations. Therefore, the presence of social cues (eg, proofs and reviews from other donors) helps to exhibit the authenticity and credibility of a project, thereby inspiring a higher level of cognition-based trust from potential donors. On this basis, we posit the following hypothesis:

H6a: Potential donors’ cognition-based trust in a medical crowdfunding website with social cues is higher than that without social cues.

The presentation of social cues on a website leads to a perception of warmth and friendliness, which triggers a sense of social presence [79]. Given that social presence is strongly associated with emotional responses and interpersonal warmth [80,81], the use of social cues will help users build affect-based trust [79]. Furthermore, affect-based trust also indicates that the trustor builds trust in the trustee according to their benevolence and moral norms [82]. In medical crowdfunding websites, social cues such as other donors’ feedback and the fundraiser’s replies can be used as third-party opinions to evaluate the fundraiser’s benevolence and moral norms, which enhance affect-based trust-building. Thus, we posit the following hypothesis:

H6b: Potential donors’ affect-based trust in a medical crowdfunding website with social cues is higher than that without social cues.

**Figure 1:**
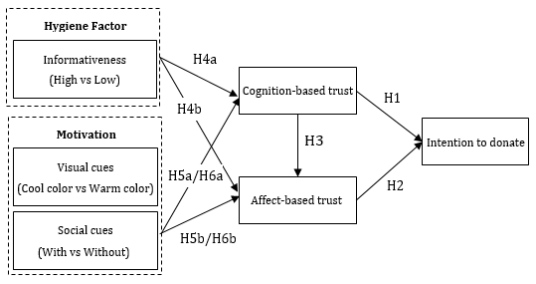
Research model.

On the basis of these hypotheses, we propose the research model shown in Figure 1.

## Methods

### Experimental Design

To test how the design features of medical crowdfunding websites influence potential donors’ intention to donate, we performed a laboratory experiment with a 2 (informativeness: high vs low) × 2 (visual cues: cool color vs warm color) × 2 (social cues: with vs without) between-subject factorial design to test the hypotheses. We designed eight medical crowdfunding webpages for the experiment.

The webpages were developed to mimic the layout of the mobile versions of Qingsongchou, a major medical crowdfunding platform in China, to enhance the external validity. A fictional medical crowdfunding project was shown in these webpages. The project began with a charity appeal about a patient with cancer who cannot pay his medical bills. We chose cancer for two reasons. First, cancer is one of the most common disease types with a high mortality rate. Second, we crawled the information of medical crowdfunding projects on the Qingsongchou website, and found that cancer is the most common cause in medical crowdfunding projects. To protect patient privacy, we made changes to basic patient information (eg, profile picture, nickname, name, and home address). Eight screenshots of the experimental webpages are presented in Multimedia Appendix 1.

### Variable Manipulations

#### Approach

We used a laboratory experimental approach because it allowed us to examine the effects of website design features on users’ intention to donate. Three website design features needed to be manipulated in our study: informativeness, visual cues, and social cues.

#### Manipulations of Informativeness

This study manipulated the informativeness of the medical crowdfunding webpages into two groups: high informativeness and low informativeness. The high informativeness group presented more categories of information (ie, patient’s charity appeal, goals and progress of crowdfunding, medical certificate, proof of the patient’s financial hardship, and information about the disease) in the webpage, along with more pictures and longer text. The low informativeness group contained fewer categories of information (ie, only the patient’s charity appeal, goals and progress of crowdfunding, and medical certificate), along with fewer pictures and shorter text.

#### Manipulations of Visual Cues

The visual cues in this study focused on the overall color of the webpages, including the background color of the pages and the font colors. The main color of the pages in the cool color groups was blue (hue: 170, saturation: 239, luminance: 128, transparency: 90%), whereas the main color of the warm color groups was orange (hue: 15, saturation: 239, luminance: 128, transparency: 90%) [83].

#### Manipulations of Social Cues

Webpages in the “with social cues” group showed the buttons for retweeting the project to social media, social endorsement from others, and information about other people’s donations. Those in the “without social cues” groups did not contain such information.

### Measurement

This research model includes three latent constructs: cognition-based trust, affect-based trust, and intention to donate. We selected the scales of these constructs from previous research with adaptive modifications to fit the context of medical crowdfunding. All of the measurements were scored on a 7-point scale (1: absolutely disagree, 7: absolutely agree). To eliminate the influence of the participants’ mood on the results of the experiment, we introduced participants’ mood as a covariate in the research model. Mood was measured by the item “How would you rate your current emotional state?”, which was scored from 1 (negative) to 7 (positive). The scale showed good reliability and validity.

We used the backtranslation method to translate the original English scale into the Chinese version and compared the two versions of the scales to check for translation accuracy. Four experts in the field of information systems were invited to evaluate the content validity of the scales. Following the opinions of the survey experts, we made some revisions to the items, such as revising the wording to improve clarity and accuracy, shortening the length of items to reduce survey fatigue, modifying ambiguous items, and revising items to make them more tailored to the medical crowdfunding context. Table 3 shows the three latent constructs and their measurement items.

**Table 3:** Research constructs and measurements.

Construct	Measurement items	References
Cognition-based trust	(1) I can rely on this medical crowdfunding platform and project; (2) the medical crowdfunding platform and project have my confidence; (3) the medical crowdfunding platform and project have high integrity	Kang et al [18], Ranganathan et al [84]
Affect-based trust	(1) If I share my problems with this medical crowdfunding platform, it would respond caringly; (2) the medical crowdfunding platform displays a warm and caring attitude toward me; (3) I can talk freely with the medical crowdfunding platform about my problems	Ranganathan et al [84], Johnson and Grayson [85]
Intention to donate	(1) The probability that I would donate money to the medical crowdfunding project is high; (2) my donation intention to the medical crowdfunding project is high; (3) the likelihood of my donating money to the medical crowdfunding project is high	Liu et al [15]

### Data Collection

A total of 320 undergraduate students recruited from a university in China participated in the controlled laboratory experiment. Each participant was assigned to one of eight webpages varying in informativeness, color, and social cues. The participants were then asked to complete the questionnaire after viewing the webpage. The age of the participants ranged from 18 to 26 years (mean 21.719 years). Analysis of variance showed no significant differences in gender (*F*_7,312_=1.097, *P*=.37) or age (*F*_7,312_=1.690, *P*=.11) across the eight experimental conditions. All subjects were randomly assigned to one of these eight treatment groups and completed the questionnaire after viewing their assigned webpages.

## Results

### Manipulation Checks

We performed manipulation checks using the measurement items adapted from previous studies. The item “The medical crowdfunding platform offers relevant, timely, and accurate information about the project” was adopted from the works of Kang et al [18] and Kim et al [86] to measure informativeness. This item was scored on a 7-point Likert scale (1: absolutely disagree, 7: absolutely agree). The item “To what extent do you feel warm when you see the colors of the website’s interface?” was adopted from the work of Motoki et al [87] to measure the warmth of the webpage’s color, which was also rated on a 7-point Likert scale (1: not warm at all to 7: very warm). We also checked the presence of social cues by adapting the scale of Friedrich et al [88], “Have you noticed the social cues on the medical crowdfunding site?”, with the item rated on a 3-point scale (1=no, 2=not sure, 3=yes).

The results from a *t* test revealed that subjects assigned to the high informativeness conditions agreed more that the webpage has richer information than those in low informativeness conditions (mean 4.925 vs 3.031, *t_318_*=17.618; *P*<.001). Similarly, the *t* test results showed that the participants who viewed webpages with orange color felt warmer than those who viewed pages with blue as the dominant color (mean 4.875 vs 2.663, *t_318_*=18.829; *P*<.001). The *t* test results also suggested that the score for the webpages with social cues was higher than that of pages without social cues (mean 2.838 vs 1.250, *t_318_*=29.088; *P*<.001). All of the results of the manipulation check were significant.

### Measurement Validation

Cronbach α for checking the internal consistency was initially computed using SPSS 24.0. Table 4 shows that the Cronbach α of each construct exceeded .90, which is higher than the recommended value of .70. A confirmatory factor analysis was then performed using AMOS 22.0 to examine the convergent validity of the constructs. Table 4 shows that the values of factor loadings and composite reliability of all constructs were greater than 0.70, and the average variance extracted (AVE) exceeded 0.5. These results showed good convergent validity [89].

**Table 4:** Convergent validity and internal reliability.

Construct			Factor loading		Cronbach α		AVE^a^		CR^b^
**Cognition-based trust (CT**)					.909		0.773		0.911
	CT1	0.814							
	CT2	0.907							
	CT3	0.913							
**Affect-based trust (AT)**					.865		0.686		0.867
	AT1	0.780							
	AT2	0.848							
	AT3	0.854							
**Intention to donate (ID)**					.915		0.784		0.916
	ID1	0.881							
	ID2	0.903							
	ID3	0.872							

^a^AVE: average variance extracted.

^b^CR: construct reliability.

Finally, we computed the square root of the AVE values of all constructs and the correlation coefficients between these constructs to test the discriminant validity. The square roots of the AVE values were greater than other correlation coefficients, which indicated adequate discriminant validity [90].

### Hypothesis Testing

We utilized AMOS22.0 to test the hypotheses regarding the effects of cognition-based trust and affect-based trust on intention to donate. As predicted, cognition-based trust had a significant and positive influence on intention to donate (β=.528, *P*<.001), supporting H1. The effect of affect-based trust on intention to donate was significant and positive (β=.344, *P*<.001), supporting H2. In addition, cognition-based trust significantly influenced affect-based trust (β=.641, *P*<.001), supporting H3. Thus, H1-H3 were supported.

Given that we have two dependent variables (ie, cognition-based trust and affect-based trust), we performed multivariate analysis of variance to check whether the experimental treatments had a general effect on both variables. Three website design features (ie, informativeness, visual cues, and social cues) were modeled as the fixed factors, and participants’ mood was included as a covariate. The main effects of informativeness (Wilks λ=0.862, *F*_310_=24.870; *P*<.001), visual cues (Wilks *λ*=0.902, *F*_310_=16.846; *P*<.001), and social cues (Wilks *λ*=0.882, *F*_310_=20.741; *P*<.001) were significant. We then performed two analyses of covariance (ANCOVAs) on cognition-based trust and affect-based trust separately.

A three-way ANCOVA was initially performed on cognition-based trust. The main effect of informativeness on cognition-based trust was significant (*F*_1,311_=49.764, *P*<.001), indicating that higher informativeness is associated with higher donor cognition-based trust in the platform and the project. Thus, H4a was supported. Similarly, social cues had a significant effect on cognition-based trust (*F*_1,311_=38.160, *P*<.001). In other words, donors’ cognition-based trust is higher when social cues are present in medical crowdfunding webpages. Thus, H6a was supported. However, visual cues had no significant effect on cognition-based trust (*F*_1,311_=1.707, *P*=.19). Thus, H5a was not supported. Moreover, no significant interaction effect was observed among informativeness, visual cues, and social cues.

A three-way ANCOVA was then performed on affect-based trust. The results indicated a significant main effect of informativeness (*F*_1,311_=16.093, *P*<.001), visual cues (*F*_1,311_=17.120, *P*<.001), and social cues (*F*_1,311_=23.265, *P*<.001). Thus, H4b, H5b, and H6b were supported. Moreover, no interaction effect was found.

### Mediation Analysis

To further verify whether the two different types of trust mediate the effects of website design features on intention to donate, we performed a mediation analysis in SPSS 24.0 with the PROCESS plugin developed by Hayes [91]. First, we used cognition-based trust as the independent variable, affect-based trust as the mediator, and intention to donate as the dependent variable. A mediation bootstrapping analysis with 5000 bootstrap samples revealed that the bootstrap 95% CI did not include 0 (β=.185, 95% CI 0.120-0.261), showing that affect-based trust mediates the effect of cognition-based trust on intention to donate.

Next, we used intention to donate as the dependent variable, three website design features (ie, informativeness, social cues, and visual cues) as independent variables, and cognition-based trust as the mediator to test the mediating mechanisms involved. The effects of informativeness (β=.545, 95% CI 0.380-0.721) and social cues (β=.454, 95% CI 0.297-0.629) on intention to donate were mediated by cognition-based trust. However, cognition-based trust did not mediate the relationship between visual cues and intention to donate (β=−.102, 95% CI –0.271 to 0.692).

Finally, we examined the mediation role of affect-based trust. Affect-based trust mediated the relationship between informativeness (β=.159, 95% CI 0.105-0.214), visual cues (β=.325, 95% CI 0.178-0.482), social cues (β=.299, 95% CI 0.141-0.464), and intention to donate.

## Discussion

### Principal Findings

This study reveals that cognition- and affect-based trust significantly influence medical crowdfunding users’ intention to donate. This finding is in line with previous studies that demonstrate the salient impact relationship between trust and intention to donate [17,19]. However, the difference is that this study did not distinguish among the different dimensions of trust [19,20,92] and instead only examined cognition-based trust [17]. This study validates the effects of different dimensions of trust on intention to donate in the context of medical crowdfunding. The results show that not only does cognition-based trust have a significant effect on intention to donate for medical crowdfunding projects but so does affect-based trust. We further found that affect-based trust plays a mediating role in the relationship between cognition-based trust and intention to donate.

Furthermore, this study introduced Hertzberg two-factor theory into the research model and identified two types of medical crowdfunding website design features (ie, hygiene and motivator factors). The results indicate that informativeness as a hygiene factor positively influences potential donors’ cognition-based trust and affect-based trust. Among the motivation factors, social cues significantly influence potential donors’ cognition-based trust and affect-based trust. However, webpage color as a visual cue affects the two dimensions of trust differently. Specifically, medical crowdfunding webpages with a warm color were more likely to induce affect-based trust than those with a cool color, whereas no significant difference was found between the effects of cool and warm colors on cognition-based trust. One possible explanation for this finding is that cognitive processes are not instantaneous, and cognition-based trust is formed only after individuals can cognitively process and evaluate available evidence [93,94]. The evidence presented on medical crowdfunding websites has a bearing on whether a patient can be cured of a serious illness and escape bankruptcy due to the illness, which requires the potential donors to devote energy to evaluate and extrapolate. Although different hues of color can cause potential donors to perceive temperature and interpersonal warmth differently, these perceptions are not directly responsible for the differences in their cognition-based trust.

Our results also indicate that cognition- and affect-based trust have mediating effects on the relationship between website design features and intention to donate. Different website design features have different influences on cognition- and affect-based trust, which in turn exert different effects on intention to donate. Particularly, informativeness and social cues indirectly affect intention to donate through cognition- and affect-based trust. Webpage colors significantly affected intention to donate through affect-based trust, whereas cognition-based trust had no mediating effect on the relationship between webpage colors and intention to donate.

### Implications

#### Theoretical Implications

The theoretical contributions of this study are as follows. First, this study contributes to the theory of trust and enriches the literature on crowdfunding. Despite the existence of impressive studies that have examined trust and willingness to fund in different crowdfunding contexts, few of them have discussed trust issues in the context of medical crowdfunding. This study identifies the importance of affect-based trust in medical crowdfunding, which is often ignored in other crowdfunding contexts. Specifically, affect-based trust not only influences intention to donate for medical crowdfunding projects simultaneously with cognition-based trust but it also mediates the influence of cognition-based trust on intention to donate.

Second, identifying the antecedents that influence cognition- and affect-based trust is important for trust development. This study explored the holistic aspects of the design features of medical crowdfunding websites to understand how users develop trust to medical crowdfunding platforms and projects. We applied Hertzberg two-factor theory to identify hygiene and motivation factors in website design features as antecedent variables that influence the two types of trust. We validated the positive influence of informativeness as a hygiene factor of website design on cognition- and affect-based trust, and demonstrated the motivating effects of visual and social cues on trust. These findings enrich the literature on website design by broadening the application of two-factor theory to the design of medical crowdfunding websites.

Third, previous studies have briefly introduced the relationships between website design features and users’ behavioral intention; however, few studies have attempted to reveal the mechanisms underlying the effects of website design features on the intention to donate in medical crowdfunding. By integrating the design features of medical crowdfunding websites, trust, and intention to donate, we contribute to filling this gap, while demonstrating the mediating role of cognition-based trust and affect-based trust on such impacts.

#### Practical Implications

This study has important implications for medical crowdfunding platform operators and users. First, our empirical results reveal the significant effects of cognition- and affect-based trust on intention to donate. Therefore, platform operators and project initiators of medical crowdfunding should not only enhance the authenticity and credibility of their platforms and projects but also emphasize their affective elements.

Second, the informativeness of the projects presented on medical crowdfunding platforms is a hygiene factor that can elicit users’ trust. Medical crowdfunding platform operators should develop more functions that contribute to the information richness of the website, such as providing a wider range of information categories and the functions of uploading pictures and videos. Moreover, project initiators need to elaborate more on their situations and charitable appeals to inspire potential donors logically and emotionally.

Third, visual and social cues on medical crowdfunding platforms also have significant effects on potential donors’ trust. This finding provides medical crowdfunding website designers with some guidelines. They can enhance the usage of warm colors to create a warm and caring atmosphere to boost users’ affect-based trust. They can also provide more social cues such as other peoples’ comments to increase potential donors’ cognition- and affect-based trust.

### Limitations and Future Research Directions

Despite the contributions of this study, we have identified several limitations. First, the subjects in this experiment were undergraduate students. Our primary reason for choosing students as subjects was that students are easier to recruit than other social groups and are more obedient, which is beneficial to the success of laboratory experiments. In addition, as Chinese medical crowdfunding projects are mainly diffused through internet apps such as Weibo and WeChat, social media users have become the main source of donations. Weibo users under the age of 25 account for 57.4% of the total number of users [95]. In terms of age distribution, students are an important user group for social media and are thus the main potential donors for medical crowdfunding. However, despite the representativeness of students, they still do not accurately represent the overall population of medical crowdfunding users. Therefore, future research will need to recruit a larger population to improve the representativeness of the subjects.

Second, this study focused on three website design features based on the previous literature and the context of medical crowdfunding. However, there may be other website design features that can influence users’ trust and donations. For example, we only focused on color in visual cues, whereas font styles or page layout may also affect users’ trust in the platform and the project. Future research can identify more website design features of relevance.

Third, this study manipulated informativeness as a one-dimensional variable incorporating text length, number of pictures, and categories of information without considering the interactions among these features. Future research can introduce new content-related features and further explore the interactions among these factors.

Finally, individuals may perceive colors in varied and nuanced ways. In our study, we only focused on one color feature that is most likely to be noticed by the user (ie, hue) and ignored the other features of color. Future research can explore the effects of other color features (eg, saturation and luminance) on potential donors’ trust.

### Conclusions

The purpose of this study was to explore the role of cognition- and affect-based trust in the medical crowdfunding context and to test the effects of medical crowdfunding website design features on these two dimensions of trust. By applying trust theory and Hertzberg two-factor theory, we identified several hygiene and motivation website design factors, and confirmed their influencing mechanisms on trust in our empirical study. This study not only enriches the literature on crowdfunding but also provides implications for medical crowdfunding platform operators and users on how to promote trust.

## Appendix

Multimedia Appendix 1 Eight screenshots of the experimental webpages.

## Abbreviations

ANCOVA: analysis of covariance
AVE: average variance extracted

## References

[1] Aizenman N (2017). Health care costs push a staggering number of people into extreme poverty. National Public Radio.

[2] Konard W (2016). Health care costs still push Americans into poverty. CBS News.

[3] Huang ZL, Shan J (2017). China aims at poverty caused by medical bills. China Daily.

[4] Reuters (2016). China healthcare costs forcing patients into crippling debt. Fortune.

[5] Burtch G, Chan J (2019). Investigating the relationship between medical crowdfunding and personal bankruptcy in the United States: evidence of a digital divide. MIS Quart.

[6] Hunt J (2019). Crowdfunding strategies for medical expenses. The Balance.

[7] Beldad A, Gosselt J, Hegner S, Leushuis R (2014). Generous but not morally obliged? Determinants of Dutch and American Donors’ Repeat Donation Intention (REPDON). Voluntas.

[8] Ebrahim A, Renz DO, Herman RD (2010). The many faces of nonprofit accountability. The Jossey-Bass handbook of nonprofit leadership and management. 3rd edition.

[9] Heath S (2020). Does medical crowdfunding perpetuate affordability problems?. Patient Engagement HIT.

[10] Berliner LS, Kenworthy NJ (2017). Producing a worthy illness: Personal crowdfunding amidst financial crisis. Soc Sci Med.

[11] Gleasure R, Feller J (2016). Does heart or head rule donor behaviors in charitable crowdfunding markets?. Int J E Commerce.

[12] Kim MJ, Bonn M, Lee C (2019). The effects of motivation, deterrents, trust, and risk on tourism crowdfunding behavior. Asia Pacif J Tourism Res.

[13] Majumdar A, Bose I (2018). My words for your pizza: An analysis of persuasive narratives in online crowdfunding. Inf Manage.

[14] Mollick E (2014). The dynamics of crowdfunding: An exploratory study. J Bus Ventur.

[15] Liu L, Suh A, Wagner C (2018). Empathy or perceived credibility? An empirical study on individual donation behavior in charitable crowdfunding. Internet Res.

[16] Agrawal A, Catalini C, Goldfarb A (2015). Crowdfunding: geography, social networks, and the timing of investment decisions. J Econ Manage Strateg.

[17] Zheng H, Hung J, Qi Z, Xu B (2016). The role of trust management in reward-based crowdfunding. Online Inf Rev.

[18] Kang M, Gao Y, Wang T, Zheng H (2016). Understanding the determinants of funders’ investment intentions on crowdfunding platforms. Indust Manag Data Syst.

[19] Liang T, Wu SP, Huang C (2019). Why funders invest in crowdfunding projects: Role of trust from the dual-process perspective. Inf Manage.

[20] Moysidou K, Hausberg JP (2019). In crowdfunding we trust: A trust-building model in lending crowdfunding. J Small Bus Manag.

[21] Strohmaier D, Zeng J, Hafeez M (2019). Trust, distrust, and crowdfunding: A study on perceptions of institutional mechanisms. Telemat Informat.

[22] Kemp E, Kennett-Hensel PA, Kees J (2013). Pulling on the heartstrings: examining the effects of emotions and gender in persuasive appeals. J Advertising.

[23] Wang W, Qiu L, Kim D, Benbasat I (2016). Effects of rational and social appeals of online recommendation agents on cognition- and affect-based trust. Decis Support Syst.

[24] McAllister DJ (1995). Affect- and cognition-based trust as foundations for interpersonal cooperation in organizations. Acad Manag J.

[25] Chen Y, Dai R, Yao J, Li Y (2019). Donate time or money? The determinants of donation intention in online crowdfunding. Sustainability.

[26] Cascino S, Correia M, Tamayo A (2019). Does consumer protection enhance disclosure credibility in reward crowdfunding?. J Account Res.

[27] Zhang Y, Tan CD, Sun J, Yang Z (2020). Why do people patronize donation-based crowdfunding platforms? An activity perspective of critical success factors. Comput Hum Behav.

[28] Behl A, Dutta P, Sheorey P, Singh RK (2020). Examining the role of dialogic communication and trust in donation-based crowdfunding tasks using information quality perspective. TQM J.

[29] Kim MJ, Hall CM, Kim D (2020). Why do investors participate in tourism incentive crowdfunding? The effects of attribution and trust on willingness to fund. J Travel Tourism Market.

[30] Wehnert P, Baccarella CV, Beckmann M (2019). In crowdfunding we trust? Investigating crowdfunding success as a signal for enhancing trust in sustainable product features. Technol Forecast Soc Change.

[31] Rodriguez-Ricardo Y, Sicilia M, López M (2019). Altruism and internal locus of control as determinants of the intention to participate in crowdfunding: the mediating role of trust. J Theor Appl Electron Commer Res.

[32] Yang X, Zhao K, Tao X, Shiu E (2019). Developing and validating a theory-based model of crowdfunding investment intention—perspectives from social exchange theory and customer value perspective. Sustainability.

[33] Jones H, Moncur W (2020). A mixed-methods approach to understanding funder trust and due diligence processes in online crowdfunding investment. Trans Soc Comput.

[34] Xiao L (2019). How lead investors build trust in the specific context of a campaign. Int J Entrepren Behav Res.

[35] Klement F, Teubner T (2019). Trust isn’t blind: exploring visual investor cues in equity crowdfunding.

[36] Lewis JD, Weigert A (1985). Trust as a social reality. Soc Forces.

[37] Punyatoya P (2019). Effects of cognitive and affective trust on online customer behavior. Market Intell Plan.

[38] Greenberg M, Gerber E (2014). Learning to fail: experiencing public failure online through crowdfunding.

[39] Moore B (2019). Medical crowdfunding and the virtuous donor. Bioethics.

[40] Kanawattanachai P, Yoo Y (2002). Dynamic nature of trust in virtual teams. J Strat Inf Syst.

[41] Xu K, Wang X (2020). "Kindhearted people, please save my family": narrative strategies for new media medical crowdfunding. Health Commun.

[42] Hsu M, Chang C, Yen C (2011). Exploring the antecedents of trust in virtual communities. Behav Inf Technol.

[43] Mpinganjira M (2018). Precursors of trust in virtual health communities: A hierarchical investigation. Inf Manag.

[44] Nadeem W, Khani AH, Schultz CD, Adam NA, Attar RW, Hajli N (2020). How social presence drives commitment and loyalty with online brand communities? the role of social commerce trust. J Retail Consum Serv.

[45] Shankar V, Urban GL, Sultan F (2002). Online trust: a stakeholder perspective, concepts, implications, and future directions. J Strateg Inf Syst.

[46] Pengnate S, Sarathy R (2017). An experimental investigation of the influence of website emotional design features on trust in unfamiliar online vendors. Comput Hum Behav.

[47] Ganguly B, Dash SB, Cyr D (2009). Website characteristics, trust and purchase intention in online stores:-an empirical study in the Indian context. J Inf Sci Technol.

[48] Cyr D (2014). Modeling web site design across cultures: relationships to trust, satisfaction, and e-loyalty. J Manag Inf Syst.

[49] Frik A, Mittone L (2019). Factors influencing the perception of website privacy trustworthiness and users' purchasing intentions: the behavioral economics perspective. J Theor Appl Electron Commer Res.

[50] Faisal CMN, Gonzalez-Rodriguez M, Fernandez-Lanvin D, de Andres-Suarez J (2017). Web design attributes in building user trust, satisfaction, and loyalty for a high uncertainty avoidance culture. IEEE Trans Human-Mach Syst.

[51] Hart TA, Chaparro BS, Halcomb CG (2008). Evaluating websites for older adults: adherence to ‘senior-friendly’ guidelines and end-user performance. Behav Inf Technol.

[52] Deng L, Poole MS (2010). Affect in web interfaces: a study of the impacts of web page visual complexity and order. MIS Quart.

[53] Sanjeev MA, Surya AV (2016). Two factor theory of motivation and satisfaction: an empirical verification. Ann Data Sci.

[54] Wu TL, Deng ZH, Chen Z, Zhang DL, Wang RX, Wu X (2019). Predictors of patients' intention to interact with doctors in web-based health communities in China: cross-sectional study. J Med Internet Res.

[55] Zhang P, von Dran GM (2000). Satisfiers and dissatisfiers: A two-factor model for website design and evaluation. J Am Soc Inf Sci.

[56] Lo LY, Lin S, Hsu L (2016). Motivation for online impulse buying: A two-factor theory perspective. Int J Inf Manag.

[57] Lin S, Lo L (2016). Motivation for using the social commerce website in the sharing economy: a two-factor theory perspective. https://aisel.aisnet.org/pacis2016/108.

[58] Ou CX, Sia CL (2009). To trust or to distrust, that is the question. Commun ACM.

[59] Chen Q, Clifford SJ, Wells WD (2002). Attitude toward the site II: new information. J Advert Res.

[60] Ducoffe RH (1995). How consumers assess the value of advertising. J Curr Issues Res Advert.

[61] Goel L, Prokopec S (2009). If you build it will they come?—An empirical investigation of consumer perceptions and strategy in virtual worlds. Electron Commer Res.

[62] Hausman AV, Siekpe JS (2009). The effect of web interface features on consumer online purchase intentions. J Bus Res.

[63] Kwak DHA, Ramamurthy KR, Nazareth D, Lee S (2018). The moderating role of helper's high in anchoring process: An empirical investigation in the context of charity website design. Comput Hum Behav.

[64] Danish Habib M, Qayyum A (2018). Cognitive emotion theory and emotion-action tendency in online impulsive buying behavior. J Manag Sci.

[65] Cimbalo RS, Beck KL, Sendziak DS (1978). Emotionally toned pictures and color selection for children and college students. J Genet Psychol.

[66] Hawlitschek F, Jansen L, Lux E, Teubner T, Weinhardt C (2016). Colors and trust: the influence of user interface design on trust and reciprocity.

[67] Williams LE, Bargh JA (2008). Experiencing physical warmth promotes interpersonal warmth. Science.

[68] Fenko A, Schifferstein HN, Hekkert P (2010). Looking hot or feeling hot: What determines the product experience of warmth?. Mater Design.

[69] Rizomyliotis I, Konstantoulaki K, Kostopoulos I (2021). Reassessing the effect of colour on attitude and behavioural intentions in promotional activities: the moderating role of mood and involvement. Australasian Market J.

[70] Bellizzi JA, Hite RE (1992). Environmental color, consumer feelings, and purchase likelihood. Psychol Market.

[71] Baxter SM, Ilicic J, Kulczynski A (2017). Roses are red, violets are blue, sophisticated brands have a Tiffany Hue: the effect of iconic brand color priming on brand personality judgments. J Brand Manag.

[72] Ettis SA (2017). Examining the relationships between online store atmospheric color, flow experience and consumer behavior. J Retail Consum Serv.

[73] Ijzerman H, Semin GR (2009). The thermometer of social relations: mapping social proximity on temperature. Psychol Sci.

[74] Citron FMM, Goldberg AE (2014). Social context modulates the effect of physical warmth on perceived interpersonal kindness: a study of embodied metaphors. Lang Cognition.

[75] Rai D, Lin CW, Yang CM (2017). The effects of temperature cues on charitable donation. J Consum Market.

[76] Brasel SA, Gips J (2011). Red Bull “Gives You Wings” for better or worse: A double-edged impact of brand exposure on consumer performance. J Consum Psychol.

[77] Mudambi SM, Schuff D (2010). What makes a helpful online review? A study of customer reviews on Amazon.com. MIS Quart.

[78] Kim JG, Kong HK, Karahalios K, Fu WT, Hong H (2016). The power of collective endorsements: credibility factors in medical crowdfunding campaigns.

[79] Xu Q (2014). Should I trust him? The effects of reviewer profile characteristics on eWOM credibility. Comput Hum Behav.

[80] Walther JB, Slovacek CL, Tidwell LC (2016). Is a picture worth a thousand words?. Commun Res.

[81] Thabet M, Zghal M (2013). An exploratory approach to the influence of perceived social presence on consumer trust in a website. J Int E-Bus Stud.

[82] Calefato F, Lanubile F, Novielli N (2015). The role of social media in affective trust building in customer–supplier relationships. Electron Commer Res.

[83] Choi J, Li YJ, Rangan P, Yin B, Singh SN (2020). Opposites attract: Impact of background color on effectiveness of emotional charity appeals. Int J Res Market.

[84] Kumar Ranganathan S, Madupu V, Sen S, R. Brooks J (2013). Affective and cognitive antecedents of customer loyalty towards e-mail service providers. J Serv Market.

[85] Johnson D, Grayson K (2005). Cognitive and affective trust in service relationships. J Bus Res.

[86] Kim JU, Kim WJ, Park SC (2010). Consumer perceptions on web advertisements and motivation factors to purchase in the online shopping. Comput Hum Behav.

[87] Motoki K, Saito T, Nouchi R, Kawashima R, Sugiura M (2019). Light colors and comfortable warmth: Crossmodal correspondences between thermal sensations and color lightness influence consumer behavior. Food Qual Pref.

[88] Friedrich T, Schlauderer S, Overhage S (2019). The impact of social commerce feature richness on website stickiness through cognitive and affective factors: An experimental study. Electron Commerce Res Appl.

[89] Fornell C, Larcker DF (1981). Evaluating structural equation models with unobservable variables and measurement error. J Market Res.

[90] Nunnally J (1978). Psychometric theory. 2nd edition.

[91] Hayes A (2013). Introduction to mediation, moderation, and conditional process analysis: a regression-based approach.

[92] Zhao Q, Chen CD, Wang JL, Chen P (2017). Determinants of backers’ funding intention in crowdfunding: Social exchange theory and regulatory focus. Telemat Informat.

[93] Morrow JJ, Hansen M, Pearson A (2004). The cognitive and affective antecedents of general trust within cooperative organizations. J Manag Iss.

[94] Xiao Q (2021). Understanding the asymmetric perceptions of smartphone security from security feature perspective: A comparative study. Telemat Informat.

[95] (2019). Social networking industry research report in 2019 (in Chinese). Jiguang.

